# Hypoxia Helps Maintain Nucleus Pulposus Homeostasis by Balancing Autophagy and Apoptosis

**DOI:** 10.1155/2020/5915481

**Published:** 2020-09-22

**Authors:** Han-Jun Kim, Hye-Rim Lee, Hyosung Kim, Sun Hee Do

**Affiliations:** ^1^Department of Veterinary Clinical Pathology, College of Veterinary Medicine, Konkuk University, Gwangjin-gu, Seoul 05029, Republic of Korea; ^2^Department of Bioengineering, Henry Samueli School of Engineering and Applied Sciences, University of California-Los Angeles, Los Angeles, CA 90095, USA; ^3^Center for Minimally Invasive Therapeutics (C-MIT), University of California-Los Angeles, Los Angeles, CA 90095, USA

## Abstract

Intervertebral disc degeneration (IVDD) is a common cause of lower back pain. Programmed cell death (PCD) including apoptosis and autophagy is known to play key mechanistic roles in the development of IVDD. We hypothesized that the nucleus pulposus cells that make up the center of the IVD can be affected by aging and environmental oxygen concentration, thus affecting the development of IVDD. Here, we evaluated the phenotype changes and PCD signaling in nucleus pulposus cells in two different oxygen percentages (5% (hypoxia) and 20% (normoxia)) up to serial passage 20. NP cells were isolated from the lumbar discs of rats, and the chondrogenic, autophagic, and apoptotic gene expressions were analyzed during cell culture up to serial passage 20. Hypoxia significantly increased the number of autophagosomes, as determined by monodansylcadaverine staining and transmission electron microscopy. Furthermore, hypoxia triggered the activation of autophagic flux (beclin-1, LC3-II/LC3-I ratio, and SIRT1) with a concomitant decrease in the expression of apoptotic proteins (Bax and caspase-3). Despite injury and age differences, no significant differences were observed between the *ex vivo* lumbar disc cultures of groups incubated in the hypoxic chamber. Our study provides a better understanding of autophagy- and apoptosis-related senescence in NP cells. These results also provide insight into the effects of aging on NP cells and their PCD levels during aging.

## 1. Introduction

Lower back pain and disabilities resulting from intervertebral disc (IVD) degeneration are the leading causes of incapacitation in adults [1, 2]. IVD degeneration is characterized by the dehydration of the nucleus pulposus (NP), rupture of the annulus fibrosus (AF), and calcification of the vertebral endplates (EPs). NP cells play an important role in IVD development, maintenance, and degeneration, by promoting the matrix biosynthesis of other IVD cell types [3, 4], indicating that modulating their activity could be a means to treat IVD degeneration. To investigate this, the biological responses of NP cells have been analyzed under various conditions, including microenvironments with altered oxygen and glucose levels [5–8].

The NP, composed of a gel-like, aggrecan-rich extracellular matrix (ECM) and cells, is derived from the notochord and comprises the central avascular structure of the IVD [3, 9, 10]. Its most common ECM component is type II collagen; however, types VI and XI are also present in smaller quantities [11–14]. NP ECM composition is altered by various etiological factors, including aging, infection, abnormal mechanical stress, smoking, diabetes, and trauma [3, 15]. IVD aging begins with changes in the NP, and degenerative NPs are characterized by decreased water content, cytoplasmic loss, and the presence of proteoglycans in the ECM [2, 16, 17].

Autophagy is an intracellular process that delivers cytoplasmic components to autophagosomes and lysosomes to maintain homeostasis. It is a crucial biological mechanism that is involved in both physiological and pathological conditions [18, 19]. In the articular system, autophagy regulates chondrocyte maturation and promotes the survival of terminally differentiated chondrocytes under stress [20, 21]. Decreased expression of autophagic regulators has been observed in aging joints and osteoarthritis in mice and humans and is accompanied by increased chondrocyte apoptosis [20, 22, 23]. During IVD degeneration, autophagic regulation of the NP helps improve NP cell survival and phenotype maintenance by reducing apoptosis and antioxidant feedback responses [24, 25]. Increased autophagy has been reported in rat NP tissues with aging and degeneration [26]. However, previous studies were conducted in normoxic conditions (20% O_2_) or after artificial induction of oxidative stress with H_2_O_2_, rather than examining NP cells in an environment with lower oxidative stress, as in avascular tissue.

In many cell types, hypoxia induces autophagy as a protection and survival mechanism [27]. However, since the *in vivo* environment of NP cells is hypoxic compared to other tissues, it is important to observe changes in autophagic regulation in these cells under hypoxic conditions. While autophagy has profound effects on NP cell survival and phenotype maintenance, the mechanism of basal autophagy regulation in NP cells and the effects of physiological stimulation on the process are not well understood. In this study, we focused on the effects of autophagy on NP cell survival and phenotype maintenance.

Here, we analyzed the biological responses of NP cells during aging (by serial passaging up to passage 20 (p20)) and environmental stress (normoxia and hypoxia) both *in vitro* and *ex vivo*. We evaluated the transcript and protein expression levels of genes related to the NP cell phenotype, autophagy, and apoptosis in hypoxic (5% O_2_) and normoxic (20% O_2_) conditions. In addition, vertebrae from juvenile (5 weeks) and young adult (10 weeks) rats were isolated and analyzed for histological and immunohistochemical changes in the NP following injury and hypoxic culture. This work provides an increased understanding of the autophagic pathway during hypoxia and may facilitate the development of novel therapeutic strategies for the treatment of degenerative IVD disease.

## 2. Materials and Methods

### 2.1. NP Cell Isolation and Culture

Five 5-week-old male Sprague–Dawley (SD) rats (average weight: 130 g) were obtained from Orient Bio (Seongnam, Korea). All experimental protocols were approved by the Institutional Animal Care and Use Committee of Konkuk University (Seoul, Korea) under permit numbers KU13116 and KU14075. Under sterile conditions, gel-like NP tissues were separated from the IVDs. NP tissues were pooled, vortexed, and washed twice with phosphate-buffered saline (PBS; Gibco, Carlsbad, CA, USA) and twice in *α*-minimum essential medium (*α*-MEM; Gibco) supplemented with 20% fetal bovine serum (FBS; Gibco) and 1% penicillin-streptomycin (Gibco) [28–30]. Pooled NP cells were divided into two groups (normoxic culture and hypoxic culture) at p0 and cultured in *α*-MEM at 37°C in a controlled environment (triplicates for each group, 6 dishes in total). Each cell culture dish was maintained independently throughout the experiment. Control NP cells (C-NPs) were cultured under normoxic conditions (20% O_2_, 5% CO_2_), while hypoxic NP cells (H-NPs) were cultured under hypoxic conditions (5% O_2_, 5% CO_2_) in hypoxia chambers (STEMCELL Technology, Vancouver, BC, Canada) [31, 32]. Cells were grown to 70–80% confluence in 100 mm culture dishes. To analyze responses to aging in hypoxia and normoxia, NP cells were cultured until p20 (up to 60 days) [33, 34] and harvested at p5, p15, and p20. In addition, we set 3MA-treated groups (3MA-treated C-NP and 3MA-treated H-NP) to examine the effects of the autophagy pathway in hypoxia. Both C-NP and H-NP cells were treated with 5 mM of 3MA (an inhibitor of autophagy; Sigma-Aldrich, St. Louis, MO, USA) throughout the experimental period [35, 36].

### 2.2. Cell Viability

The effects of various culture conditions on NP cell viability were determined using the 3-(4,5-dimethylthiazol-2-yl)-2,5-diphenyl-tetrazolium bromide (MTT) viability assay using a commercial kit (Roche, Basel, Switzerland). Briefly, NP cells (p5) were seeded into 96-well plates (2 × 10^2^ cells/*μ*L) and cultured for 24, 72, and 96 h in hypoxia or normoxia. MTT labeling solution was added to each well, and cells were incubated for an additional 4 h in hypoxia or normoxia. After dissolving the released formazan dye in dimethyl sulfoxide, the absorbance was measured at 595 nm using a Sunrise™ microplate reader (TECAN, Salzburg, Austria).

### 2.3. Morphometric Analysis

NP cells in a specific passage (p5, p20) were seeded on Lab-Tek chamber slides (1 × 10^2^ cells/*μ*L; Nunc, Rochester, NY, USA), cultured for 72 hours, and stained with Alizarin Red S (Sigma-Aldrich) to visualize mineralization during aging and hypoxia. The slides were fixed in cold methanol (Merck, Darmstadt, Germany) and then stained with 2% Alizarin Red S for 5 min at room temperature. Slides were then dehydrated with a graded series of acetone (Merck) and acetone : xylene (1 : 1; BBC, Mount Vernon, WA, USA) [37]. Stained monolayers were visualized by phase microscopy using an inverted microscope (Leica Microsystems, Wetzlar, Germany). Extracellular calcium deposits were indicated by bright orange-red staining.

### 2.4. Real-Time Polymerase Chain Reaction (RT-PCR)

Total RNA was extracted from cells at p5, p15, and p20 using RNAiso Plus (TaKaRa, Shiga, Japan) according to the manufacturer's instructions. Isolated total RNA (1 *μ*g) was reverse transcribed into complementary DNA (cDNA) and used in quantitative (qRT-PCR) assays using the SYBR® Green PCR Kit (Qiagen, Valencia, CA, USA) in a Rotor-Gene Real-Time PCR-Cycler® (Qiagen). The reactions (20 *μ*L) comprised 2 *μ*L of diluted cDNA, 2 *μ*L of each primer, 10 *μ*L of 2x SYBR® Green Master Mix, and 6 *μ*L of RNase-free water. The sequences of the oligonucleotide primers used in the qPCR assays are shown in Table 1. Thermocycling conditions were as follows: 50°C for 2 min, 95°C for 15 min, then 40 cycles of 94°C for 15 s, 55°C for 30 s, and 72°C for 5 min. All samples were assayed in duplicate, and mRNA levels were calculated using the 2^−*ΔΔ*Ct^ method.

### 2.5. Immunoblot Analysis

NP cells at p5, p15, and p20 were homogenized in radioimmunoprecipitation buffer containing a protease and phosphatase inhibitor cocktail (ThermoFisher Scientific, Waltham, MA, USA). Subsequently, the lysates were centrifuged at 13,572 × *g* for 10 min at 4°C to obtain soluble protein. Protein concentrations were determined by the Bradford method. Proteins of interest were immunoblotted using 35 *μ*g of protein, following standard protocols. The extracted proteins were resolved by 8–15% sodium dodecyl sulfate-polyacrylamide gel electrophoresis and transferred onto polyvinylidene difluoride membranes (Millipore, Billerica, MA, USA). After blocking with 3% bovine serum albumin (Sigma-Aldrich), the membranes were incubated with antibodies against *β*-actin (sc-130656; 1 : 200), full-length caspase-3 (sc-7272; 1 : 200), polyclonal rabbit anti-beclin-1 (ab55878; 1 : 1000; Abcam, Cambridge, UK), light chain 3 (LC3; ab58610; 1 : 1000), Bcl-2, apoptosis regulator (Bcl-2; sc-492; 1 : 200), Bcl-2 associated X, apoptosis regulator (Bax; sc-526; 1 : 200), and sirtuin 1 (SIRT1; sc-15404; 1 : 200). All antibodies were from Santa Cruz Biotechnology (Heidelberg, Germany) except the beclin-1 antibody (Abcam (Cambridge, UK)). Specific binding was detected using the Super Signal West Dura Extended Duration Substrate (ThermoFisher) and a LAS 4000 chemiluminescent image analyzer (Fujifilm, Tokyo, Japan). Protein band intensities were quantified using the ImageJ software (https://imagej.nih.gov/ij/download.html; National Institutes of Health, Bethesda, MD, USA).

### 2.6. Monodansylcadaverine (MDC) Staining

MDC staining has been used to monitor autophagy by staining autophagic vacuoles [38]. Specific passage numbers of NP cells (p5, p15, and p20) were seeded on Lab-Tek chamber slides in the same way as the morphometric analysis. Subsequently, autophagic vacuoles were labeled with MDC by incubating the cells with 0.05 mM MDC in *α*-MEM (Gibco) at 37°C for 60 min. MDC-stained autophagic vacuoles were examined using a fluorescence microscope (BX61; Olympus, Tokyo, Japan) [38, 39]. The MDC-positive cells were calculated by counting cells from at least three random microscopic fields using the ImageJ software (https://imagej.nih.gov/ij/download.html; National Institutes of Health).

### 2.7. Transmission Electron Microscopy (TEM)

NP cells cultured to p5, p15, and p20 were fixed with 4% glutaraldehyde (Sigma-Aldrich) overnight, postfixed in 2% osmium tetroxide, dehydrated with a graded series of ethanol (Merck), and embedded in resin. Images of autophagosomes were captured using a JEM 1010 transmission electron microscope (JEOL, Peabody, MA, USA). Based on the previous studies, a vacuole structure with a double to multimembranous structure in the cytoplasm was defined as an autophagosome [39, 40]. In each group (C-NP and H-NP) with specific passage (p5, p15, and p20), double membranous autophagosomes present in the cytoplasm were quantified in at least three different samples using the ImageJ software.

### 2.8. Ex Vivo Analysis Using a Disc Microinjection Organ Culture Model

Male SD rats aged 5 weeks (young, Y) and 10 weeks (old, O) were purchased from Orient Bio. Rats were housed at 22 ± 2°C with a 12 h light-dark cycle. Food (PMI Nutrition International, St. Louis, MO, USA) and water were supplied *ad libitum*. Rats were divided into four groups (*n* = 3/group): 5 weeks old without injury, 5 weeks old with injury, 10 weeks old without injury, and 10 weeks old with injury. In the injured groups, 10 *μ*L of PBS was injected into the vertebral discs with a 26-gauge needle [39, 41]. The vertebrae were dissected and removed from the rats, and the discs between the L1-L2 and L3-L4 lumbar vertebrae were separated from neighboring vertebrae using a scalpel. The isolated discs were maintained for 14 days in *α*-MEM containing 10% FBS at 37°C in a hypoxic condition (5% O_2_) and then subjected to morphometric analysis.

### 2.9. Histopathology and Immunohistochemistry

Lumbar discs were fixed in 10% neutral-buffered formalin, decalcified in Solution Lite (Sigma-Aldrich), processed using a standard method, and embedded in paraffin. Serial disc sections (4 *μ*m thick) were stained with hematoxylin and eosin (H&E) and Safranin O (Sigma-Aldrich). For immunohistochemistry, sections were subjected to heat-mediated antigen retrieval using 0.01 M sodium citrate buffer (pH 6.0). A monoclonal mouse type II collagen antibody (cp18, 1 : 100, Calbiochem, San Diego, CA, USA) was used as the primary antibody. Antigen-antibody complexes were visualized using the avidin–biotin–peroxidase complex solution from the VECTASTAIN® Avidin-Biotin Complex Staining Kit (Vector Laboratories, Burlingame, CA, USA) along with 3,3′-diaminobenzidine (Vector Laboratories). Sections were counterstained with Mayer's hematoxylin.

### 2.10. Protein Extraction from Paraffin-Embedded Tissues

Proteins were extracted from formalin-fixed, paraffin-embedded (FFPE) lumbar disc tissues using the Qproteome FFPE Tissue Kit (Qiagen, Hilden, Germany). Protein samples were combined with a polyclonal rabbit LC3 antibody (Abcam) and incubated for 2 h at 4°C. Protein A/G PLUS-Agarose beads (20 *μ*L; Santa Cruz Biotechnology) were added, and the mixtures were incubated at 4°C on a rocker platform for 2 h. The pellets were washed three times, and the buffer was carefully aspirated to avoid disturbing the pellets. Thereafter, the pellets were resuspended in 40 *μ*L of sample buffer. The immunoprecipitated samples were subjected to immunoblot analysis as described above [42].

### 2.11. Statistical Analysis

Statistical analyses were performed using GraphPad Prism 4.02 (GraphPad Software, San Diego, CA, USA). Multiple comparisons were analyzed using the one-way analysis of variance followed by the Bonferroni post hoc test. All results are expressed as the mean ± standard deviation. *p* < 0.05 was considered statistically significant.

## 3. Results

### 3.1. Effects of Hypoxia and Serial Passaging on NP Cell Viability and Mineral Accumulation

**Figure 1:**
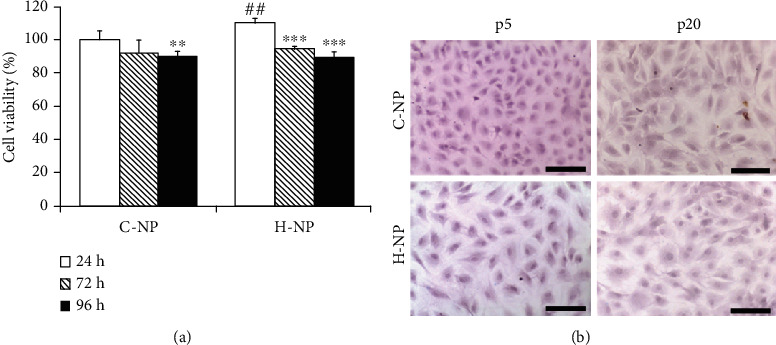
Effect of hypoxia on nucleus pulposus (NP) cell viability. (a) Viability was evaluated using the 3-(4,5-dimethylthiazol-2-yl)-2,5-diphenyl-tetrazolium bromide (MTT) assay. Data are presented as the mean ± standard deviation. (b) Alizarin Red S staining to assess morphological changes in hypoxic and aging conditions. Scale bar = 100 *μ*m. ^∗^*p* < 0.05, ^∗∗^*p* < 0.01, and ^∗∗∗^*p* < 0.001 versus 24 h; ^#^*p* < 0.05, ^##^*p* < 0.01, and ^###^*p* < 0.001 versus C-NP cells.

To assess the effects of different environmental oxygen conditions on cell viability, NP cells were cultured for 96 h in normoxic and hypoxic conditions. Interestingly, at the earliest timepoint (24 h), the viability of the hypoxic H-NP cells was significantly higher than that of the normoxic C-NP cells (*p* < 0.01). However, after 72 h, both C-NP and H-NP cells displayed slight but insignificant decreases in viability (Figure 1(a)). Next, we cultured C-NP and H-NP cells up to p20 and stained them with Alizarin Red S to examine changes in cell shape and mineralization. As shown in Figure 1(b), mineralization plaques were observed in neither H-NP nor C-NP cells until p20. However, H-NP cells were larger than C-NP cells, with increased cytoplasm, and C-NP cells were more spindle-shaped than H-NP cells at p20. These results indicate that hypoxia does not affect NP cell viability but may affect their phenotype, changing their size and morphology.

### 3.2. Chondrogenesis-Related Gene Expression in NP Cells under Different Oxygen Concentrations

**Figure 2:**
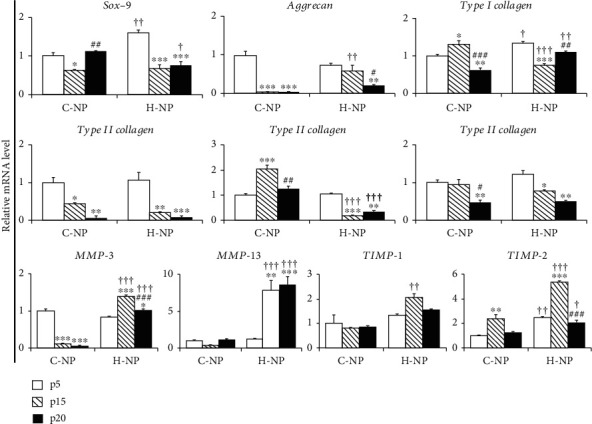
Chondrogenesis-related gene expression analysis in NP cells under different oxygen concentrations. RT-PCR was used to analyze mRNA expression levels in normoxic (C-NP) and hypoxic (H-NP) cells at p5, p15, and p20 (*n* = 3 per group). Primers targeted transcription factors (SRY-box transcription factor 9), ECM proteins (aggrecan and type I, II, III, and VI collagens), and metalloproteinase-related genes (MMP-3, MMP-13, TIMP-1, and TIMP-2). Results are the mean ± standard deviation of triplicate experiments. ^∗^*p* < 0.05, ^∗∗^*p* < 0.01, and ^∗∗∗^*p* < 0.001 versus p5; ^#^*p* < 0.05, ^##^*p* < 0.01, and ^###^*p* < 0.001 versus p15; ^†^*p* < 0.05, ^††^*p* < 0.01, and ^†††^*p* < 0.001 versus C-NP cells at the same passage number.

Given the observed morphological changes with hypoxic culture, we next sought to examine alterations of gene expressions related to chondrogenesis upon aging and hypoxia through the serial passaging of NP cells (Figure 2). This endpoint was chosen based on previous studies regarding the serial passaging of primary isolated cells to senescence [33, 34]. At the earlier passage (p5), compared to H-NP, C-NP showed downregulation of SRY-box transcription factor-9 (Sox-9), type I collagen, and tissue inhibitor of metalloproteinase-2 (TIMP-2) and slight upregulation of matrix metallopeptidase (MMP-3). After aging by serial passaging, C-NP cells exhibited significantly decreased levels of ECM-related genes (e.g., *aggrecan*, *type II collagen*, and *type VI collagen*) as well as ECM-regulating enzymes (e.g., *MMP-3*) at p15. Despite H-NP cells displayed the same tendency as C-NP cells with serial passaging (aging), the levels of ECM-related genes were significantly higher in H-NP cells compared to that in C-NP cells. The results indicate that repeated passaging under normoxia led to the dedifferentiation of NP cells, as they quickly lose aggrecan and type II collagen, while simultaneously transitioning to a fibroblastic phenotype characterized by high type III collagen expression. However, aggrecan mRNA expression at p15 was significantly higher in hypoxia (*p* < 0.01). In addition, hypoxia maintained NP cell homeostasis through increases in catabolic enzymes such as MMP-3 and MMP-13, as well as increased expression of MMP inhibitors, such as TIMP-1 and TIMP-2. These results suggest that hypoxia results in slower ECM protein degradation than normoxia and maintains homeostasis through the coordinated actions of MMPs and TIMPs.

### 3.3. Gene and Protein Levels of Autophagosome- and Autophagy-Related Genes in NP Cells under Different Oxygen Concentrations

**Figure 3:**
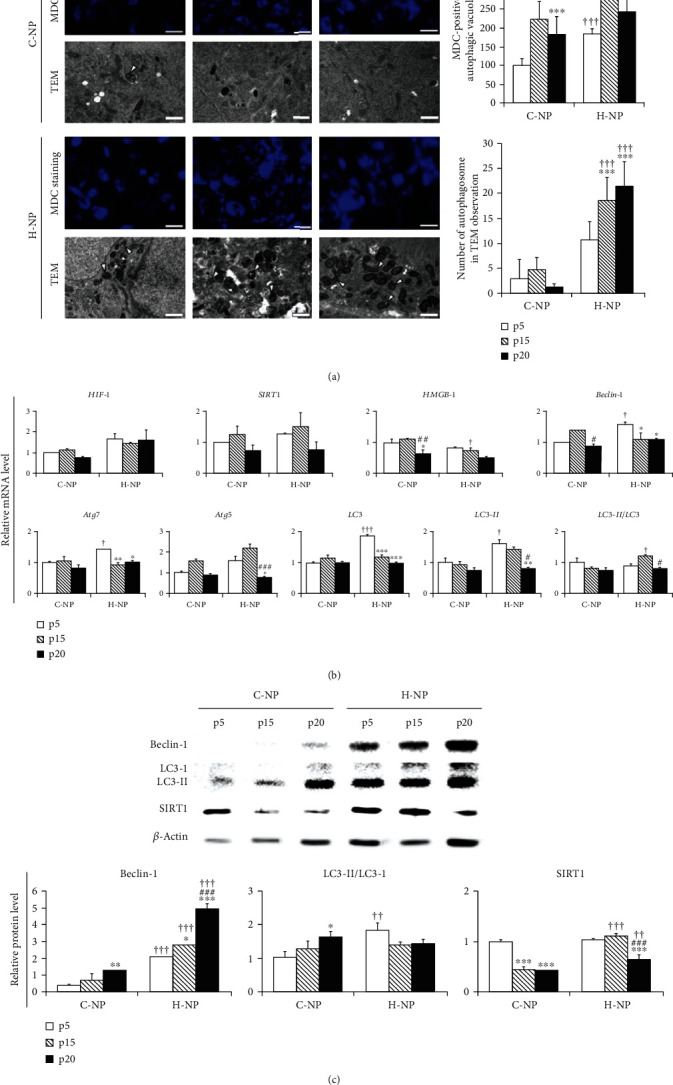
Autophagosome- and autophagy-related genes and protein level analysis in NP cells under different oxygen concentrations. (a) Representative MDC staining and TEM images and statistical analysis of autophagosomes (arrowheads) in p5, p15, and p20 NP cells. Scale bar for MDC staining: 100 *μ*m, TEM: 0.5 *μ*m. (b) RT-PCR was used to analyze mRNA expression levels in normoxic (C-NP) and hypoxic (H-NP) cells at p5, p15, and p20 (*n* = 3 per group). Primers targeted hypoxia- (HIF1, SIRT1) and autophagy- (HMGB1, beclin-1, ATG7, ATG5, LC3-I, and LC3-II) related genes. (c) Representative immunoblots and statistical analysis of protein levels in the experimental groups. Data are presented as the mean ± standard deviation. ^∗^*p* < 0.05, ^∗∗^*p* < 0.01, and ^∗∗∗^*p* < 0.001 versus p5; ^#^*p* < 0.05, ^##^*p* < 0.01, and ^###^*p* < 0.001 versus p15; ^†^*p* < 0.05, ^††^*p* < 0.01, and ^†††^*p* < 0.001 versus C-NP cells at the same passage number.

To assess the effects of hypoxia on autophagy in NP cells, the autophagic process was visualized by MDC staining and TEM (Figure 3(a)). First of all, we labeled autophagic vacuoles with MDC, a lysosomotropic agent that is incorporated into the lipids of autophagic vacuoles. Our results showed that the number of MDC-labeled autophagosomes increased with aging in both normoxic and hypoxic conditions and peaked at p15. In particular, under hypoxic conditions, the number of MDC-labeled autophagosomes was significantly higher than in normoxic conditions throughout the experimental periods. Subsequently, double membranous autophagosomes in experimental groups were analyzed using TEM. Similar to MDC staining results, H-NP cells contained more autophagosomes than C-NP cells in all passages examined. Interestingly, the difference increased with the number of passages (5.6- and 14.25-fold higher in H-NP cells vs. C-NP cells at p15 and p20, respectively).

We then investigated whether hypoxia or aging affected the transcript and protein levels of autophagy-related genes in NP cells (Figures 3(b) and 3(c)). Despite repeated passaging, there were no significant changes in the expression of autophagy-related genes in C-NP cells, except for high mobility group box 1 (HMGB1) at p20. Compared to the C-NP, H-NP showed significantly increased gene expression levels of beclin-1, autophagy-related 7 (ATG7), LC3-I, and LC3-II in the early passages (p5). At p15, the LC3-II/LC3-I ratio was significantly increased in H-NP cells compared to C-NP cells (*p* < 0.05). Consistent with this, the beclin-1 protein level (*p* < 0.001 at p5, p10, and p15) and the LC3-II/LC3-I ratio (*p* < 0.01 at p5) were significantly higher in H-NP cells. SIRT1 is a key mediator of hypoxia, which is known to promote autophagy and inhibit apoptosis to protect the cells from hypoxic stress via AMPK activation [43]. In our results, SIRT1 protein expression was significantly upregulated in H-NP cells compared to that in C-NP cells at p15 and p20. Taken together, these results suggest that NP cells have increased autophagic activation response to hypoxic condition and that this autophagic flux is related to increased beclin-1, LC3-II/LC3-I ratio, and SIRT1 activation.

### 3.4. Apoptosis and Signaling Pathway in NP Cells under Different Oxygen Concentrations

**Figure 4:**
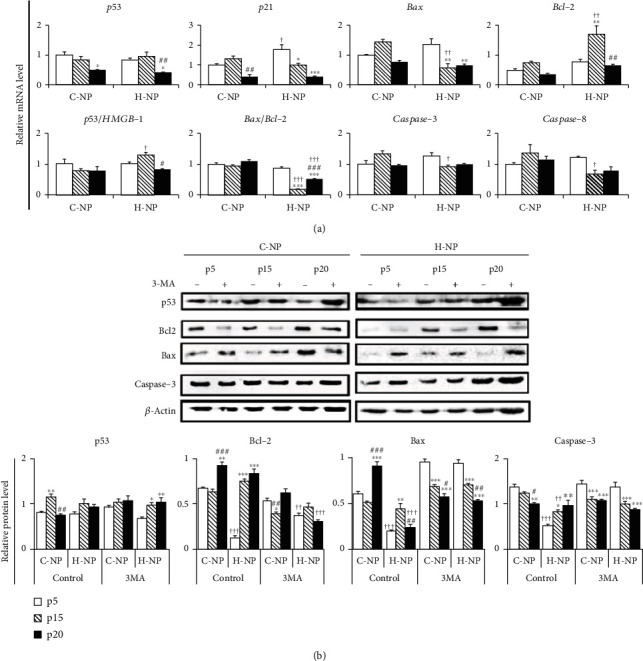
Apoptosis-related gene and protein expression analysis under the different oxygen concentrations. (a) Expression levels of apoptosis-related genes were analyzed using RT-PCR. (b) Representative immunoblots and statistical analysis of protein levels in the experimental groups. Data are presented as the mean ± standard deviation. ^∗^*p* < 0.05, ^∗∗^*p* < 0.01, and ^∗∗∗^*p* < 0.001 versus p5; ^#^*p* < 0.05, ^##^*p* < 0.01, and ^###^*p* < 0.001 versus p15; ^†^*p* < 0.05, ^††^*p* < 0.01, and ^†††^*p* < 0.001 versus C-NP cells at the same passage number.

According to previous reports [24, 25], substances developed to enhance the autophagic flux of NP cells for the treatment of IVD degeneration can reduce the activation of apoptosis-related pathways, in addition to enhancing the autophagy-related pathways. To determine whether this affects our experimental model, we analyzed the expression patterns of apoptosis-related genes and proteins (Figures 4(a) and 4(b)). Consistent with the viability results, C-NP cells did not show significant changes in apoptosis-related gene and protein expression levels with serial passaging. However, compared to C-NP cells, H-NP cells displayed increased gene expression of *Bcl-2* (*p* < 0.01) as well as decreased expression of *Bax* (*p* < 0.01), *caspase-3* (*p* < 0.05), and *caspase-8* (*p* < 0.05) at p15 (Figure 4(a)). In addition, the *Bax/Bcl-2* ratio, a measure of apoptotic susceptibility [44], was significantly lower in H-NP cells compared to that in C-NP cells (at p15 and p20, *p* < 0.001). Similar to gene expression analysis, Bax and caspase-3 protein expression levels were also significantly decreased in H-NP cells (Figure 4(b)). However, these antiapoptotic protein expressions (decreased Bax, caspase-3 expression) of NP cells under the hypoxic condition were reversed after 3MA (autophagy inhibitor) treatment. These results could indicate that hypoxic condition not only induces autophagic flux but also could exhibit antiapoptotic signaling activation in NP cells via Bax/Bcl-2 and caspase-3/8 signaling pathways.

### 3.5. Histological Changes of the Rat Lumbar Disc Ex Vivo Culture Model under the Hypoxic Condition

**Figure 5:**
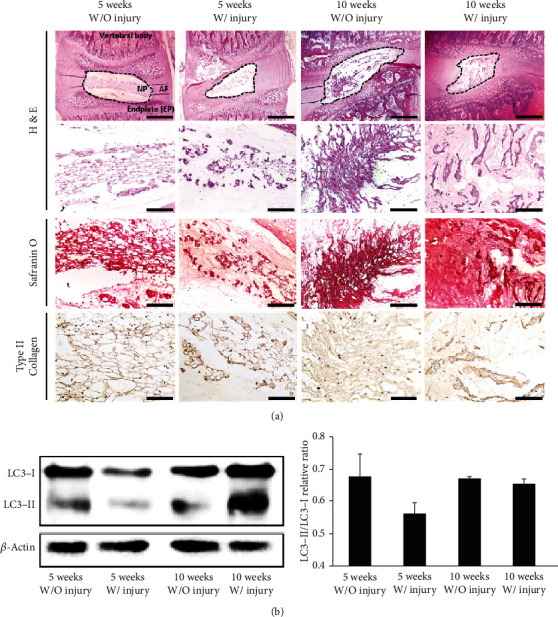
Morphometric and protein expression analyses of an *ex vivo* rat IVD culture model. (a) Representative histological images of *ex vivo* IVD cultures from 5-week-old and 10-week-old rats with or without injury (injection of 10 *μ*L of PBS into the disc). Images shown are at 40x (H&E), 200x (H&E and Safranin O), and 400x (IHC) magnification. The dotted line indicates the NP. (b) Representative immunoblotting images (left) and statistical analysis of protein levels in the experimental groups. Data are presented as the mean ± standard deviation.

Finally, we tested an ex vivo IVD culture model under the hypoxic condition as same as in vitro study. To identify changes of NP phenotype upon aging and injury, we set up a control/injury group (with/without injury) and 5-week and 10-week groups (juvenile and young adults). The no injury groups showed relatively well-preserved NP structures than the injured groups in the center of the IVD (Figure 5(a)). Nevertheless, aggregated, serpentine-shaped ECM with clustered NP cells still exist in the injured groups. In the 5 weeks without injury group, the Safranin O-positive area was homogenously distributed with the cells throughout the ECM, whereas in the 10 weeks without injury group, the cells were clustered in localized areas. Even though all 10-week-old and injured groups had ECM inside the NP area, the injured group showed condensed or degenerative features rather than homogenous distribution. These features might indicate that hypoxic conditions could help to maintain the NP cell viability; it could not cure or improve the regenerative capacity of NP cell itself. Similar to the in vitro results, the intensity of type II collagen (a major component of the NP) was higher in the juvenile groups than that in the young adult groups. The LC3-II/LC3-I ratio was higher in the 5 weeks without injury group compared with that in the 10 weeks without injury group (Figure 5(b)). Despite differences in injury and age, discs cultured in hypoxic conditions were found to exhibit a certain level of autophagic activation. These results are consistent with our in vitro TEM and qPCR/protein expression results from early passage cultures (p5).

## 4. Discussion

IVD degeneration, a major contributor to chronic lower back pain, is an age-related condition characterized by loss of the ECM and the functional cells responsible for its regeneration. The inner NP region of the vertebral disc is composed of type II collagen and proteoglycans. These molecules are responsible for water retention, which maintains the viscoelastic properties of the discs [31, 32, 41]. NP tissue is avascular, and the oxygen saturation levels required for its sustenance are relatively low compared to other tissues [35].

The microenvironment of IVD is hypoxic but not completely anaerobic (1% O_2_ in central NP), and during IVD degeneration progression, neovascularization of the disc is known to increase oxygen tension in the microenvironment of IVD [5, 6]. High oxygen tension is expected to enhance reactive oxygen species (ROS) generation and subsequently induce oxidative stress in the microenvironment of IVD, which is closely related to the establishment and progression of IVD degeneration [7, 45, 46]. In this study, we evaluated autophagy changes in relation to NP cell phenotype and apoptotic/antiapoptotic signaling during serial passaging in normoxic and hypoxic conditions.

Alizarin Red S staining revealed that although mineralization plaques were not observed until p20 in both H-NP and C-NP cells, the size and shape of the cells differed depending on the culture conditions (Figure 1(b)). RT-qPCR results demonstrated that hypoxia led to increased *aggrecan* and *type II collagen* and decreased *type III collagen* in H-NP cells. These results indicate that C-NP cells exhibit characteristics of fibrocartilage, while H-NP cells exhibited chondrogenic characteristics. In addition, hypoxia resulted in elevated levels of *TIMP-1* and *TIMP-2* (at p15), as well as expression of *MMP-3* and *MMP-13* (at p15 and p20), which regulate collagen and aggrecan degradation. Collectively, NP cells maintained a partially chondrogenic phenotype without mineralization under hypoxic conditions for 20 serial passages (approximately 60 days). The hypoxic environment plays a crucial role in maintaining the physiological function of the IVD, including cellular metabolism and matrix synthesis [15, 47]. Thus, our results indicate that the use of hypoxic conditions is important to accurately study the IVD microenvironment *in vitro*.

It is well known that apoptosis and autophagy are both closely related to the onset and progression of IVD degeneration [24, 48, 49]. Apoptosis is responsible for decreased NP cell numbers during degeneration [50–52]. Conversely, autophagy is an evolutionarily conserved process that has been implicated in cell growth, development, and stress responses [23, 40]. It is activated by various stresses, such as aberrant mechanical compression, hypoxia, high glucose, and reactive oxygen species [35, 53]. In our study, both MDC staining and TEM results showed that autophagosomes were significantly increased in the late-passage H-NP cells compared to those in the C-NP cells (Figure 3). In addition, beclin-1 expression and the LC3-II/LC3-I ratio increased, whereas Bax and caspase-3 expression decreased in H-NP cells compared with that in C-NP cells. H-NP cells were responsive to hypoxia to protect and promote autophagic influx, as indicated by increased SIRT1 expression. Autophagy is an essential protective mechanism for cell survival after injury, and SIRT1 protects cells by regulating autophagy and metabolism [54]. Furthermore, a Bcl-2/beclin-1 interaction plays a key regulatory role in autophagy, allowing Bcl-2 to inhibit both apoptosis and autophagy [55, 56]. Interestingly, both autophagic flux and antiapoptotic regulation were blocked by the autophagy inhibitor 3MA under the hypoxic condition. These results suggest that homeostasis in hypoxic conditions is promoted through both elevated autophagy and antiapoptotic effects.

There are some limitations to this study. One was the use of NP cells isolated from rat lumbar discs. Species with chondrodystrophoid discs, such as humans, sheep, and dogs, can experience profound, early-onset degenerative disc disease, which often occurs within one year of birth [11, 15]. We used rats in this study, as this model has been used in many previous studies, and rats are one of the few species that maintain an NP cell population similar to that observed in adult humans. Further studies are required to determine the relation between autophagic flux, hypoxia, and aging in humans.

In conclusion, our results provide evidence that NP cells modulate the expression of chondrogenesis-, autophagy-, and apoptosis-related genes under hypoxic conditions. This study provides a better understanding of autophagy- and apoptosis-related senescence in NP cells. These results may also provide insight into the changes that occur in NP cells during aging.

## Figures and Tables

**Table 1:** Sequences of rat primers used for real-time PCR.

Target gene	Source	Sequence	Predicted length (bp)
*GAPDH*	NM017008.4	F: AAC TCC CTC AAG ATT GTC AGC AA R: GGC TAA GCA GTT GGT GGT GC	51
*Sox-9*	NM080403.1	F: ACG GCT CCA GCA AGA ACA AG R: TTG TGC AGA TGC GGG TAC TG	109
*Aggrecan*	J03485.1	F: GAC CAG GAG CAA TGT GAG GAG R: CTC GCG GTC GGG AAA GT	72
*Type I collagen*	NM053304.1	F: TGG CCA AGA AGA CAT CCC TGA AGT R: ACA TCA GGT TTC CAC GTC TCA CCA	81
*Type II collagen*	NM012929.1	F: GAG TGG AAG AGC GGA GAC TAC TG R: CTC CAT GTT GCA GAA GAC TTT CA	81
*Type III collagen*	NM032085.1	F: TTC CTG GGA GAA ATG GCG AC R: GGC CAC CAG TTG GAC ATG AT	99
*Type VI collagen*	XM001079629.4	F: CAA GAA CAC GTG GAC ATG CG R: CAC TGC AGT TTC TTG ACG GC	77
*ALP*	NM013059.1	F: CAT GTT CCT GGG AGA TGG TA R: GTG TTG TAC GTC TTG GAG AGA	144
*Runx2*	NM001278483.1	F: GAT GAC ACT GCC ACC TCT GA R: ATG AAA TGC TTG GGA ACT GC	118
*BMP-2*	NM017178.1	F: CTA TAT GCT CGA CCT GTA CCG R: CAC TCA TTT CTG AAA GTT CCT CG	146
*TGF-β*	NM_021578.2	F: CGC AAC AAC GCA ATC TAT G R: ACC AAG GTA ACG CCA GGA	204
*TIMP-1*	NM053819.1	F: TCC CCA GAA ATC ATC GAG AC R: TCA GAT TAT GCC AGG GAA CC	250
*TIMP-2*	NM021989.2	F: CAG GGC CAA AGC AGT GAG CGA GAA R: TCT TGC CAT CTC CTT CCG CCT TCC	230
*MMP-2*	NM031054.2	F: GAT CTG CAA GCA AGA CAT TGT CTT R: GCC AAA TAA ACC GAT CCT TGA A	83
*MMP-3*	NM133523.2	F: TCC CAG GAA AAT AGC TGA GAA CTT R: GAA ACC CAA ATG CTT CAA AGA CA	74
*MMP-9*	NM031055.1	F: GTA ACC CTG GTC ACC GGA CTT R: ATA CGT TCC CGG CTG ATC AG	68
*MMP-13*	NM133530.1	F: CTG ACC TGG GAT TTC CAA AA R: ACA CGT GGT TCC CTG AGA AG	96
*HIF-1*	XM006240199.3	F: AAG TCT AGG GAT GCA GCA C R: CAA GAT CAC CAG CAT CTA G	175
*SIRT1*	XM017588054.1	F: AGC TGG GGT TTC TGT TTC CTG TGG R: TCG AAC ATG GCT TGA GGA TCT GGG A	111
*HMGB-1*	NM012963.2	F: CGG ATG CTT CTG TCA ACT TCT R: AGT TTC TTC GCA ACA TCA CCA	292
*Beclin-1*	NM001034117.1	F: TTC AAG ATC CTG GAC CGA GTG AC R: AGA CAC CAT CCT GGC GAG TTT C	142
*Atg7*	NM001012097.1	F: GAC CTG GGC TCC TCA CTT TTT G R: CCC TGG GCG GCT CAC TG	135
*Atg5*	NM001014250.1	F: AGG CTC AGT GGA GGC AAC AG R: CCC TAT CTC CCATGG AAT CTT CT	72
*LC3*	NM022867.2	F: CAT GCC GTC CGA GAA GAC CT R: GAT GAG CCG GAC ATC TTC CAC T	70
*LC3-II*	NM022867.2	F: CTT TGT AAG GGC GGT TCT R: GAG GCT TGC TTT AGT TGG	141
*p53*	NM030989.3	F: CAG CTT TGA GGT TCG TGT TTG T R: ATG CTC TTC TTT TTT GCG GAA A	82
*p21*	NM080782.3	F: CAG ACC AGC CTA ACA GAT TTC R: TGA CCC ACA GCA GAA GAA G	105
*Bax*	NM017059.2	F: CCA AGA AGC TGA GCG AGT GTC TC R: AGT TGC CAT CAG CAA ACA TGT CA	147
*Bcl-2*	NM016993.1	F: TGA ACC GGC ATC TGC ACA C R: CGT CTT CAG AGA CAG CCA GGA G	116
*Caspase-3*	NM012922.2	F: CTG GAC TGC GGT ATT GAG AC R: CCG GGT GCG GTA GAG TAA GC	104
*Caspase-8*	NM022277.1	F: TCA GCA ACA TGC GGG ACA G R: TGA AGC AGT CTT TGC CCT TGT G	171
*Caspase-9*	NM031632.1	F: GGA AGA TCG AGA GAC ATG CAG R: CCG TGA CCA TTT TCT TAG CAG	216

## Data Availability

The datasets used and/or analyzed during the current study are available from the corresponding author on reasonable request.
